# Metamaterial with Perforated Auxetic Core for Ultra-Low-Frequency Vibration Isolation of Lamb Waves

**DOI:** 10.3390/ma18122857

**Published:** 2025-06-17

**Authors:** Yating Gao, Hui Wang

**Affiliations:** 1 College of Civil Engineering, Henan University of Technology, Zhengzhou 450001, China; gaoyating@stu.haut.edu.cn; 2 School of Civil Engineering and Architecture, Hainan University, Haikou 570228, China

**Keywords:** low-frequency vibration isolation, metamaterial, local resonance, perforated auxetic core, peanut-shaped perforations

## Abstract

Low-frequency vibration isolation metamaterials (LFVIMs) remain challenging in generating ultra-low-frequency bandgaps around 10 Hz and below. For this issue, a novel LFVIM composed of a square steel auxetic core perforated with orthogonally aligned peanut-shaped holes and a silicone rubber coating is proposed, leveraging the auxetic core’s unique resonance behavior. The superiority in bandgap creation of the peanut-shaped perforations is illustrated by comparing them to elliptical and rectangular perforations. Furthermore, a filled auxetic core is explored as well, to enhance its wave attenuation potential. The wave propagation mechanisms of both the unfilled and filled LFVIMs are comparatively studied by finite element simulation validated against an existing LFVIM design and scaled-down vibration testing. Compared to the unfilled LFVIM, the filled case merges smaller bandgaps into three wider full bandgaps, increasing the relative bandgap width (RBW) from 44.25% (unfilled) to 58.93% (filled). Subsequently, the role of each design parameter is identified by parametric analysis for bandgap tuning. The coating material shows a significant influence on the RBW. Particularly, optimizing the coating’s Poisson’s ratio to 0.2 yields a maximum RBW of 93.95%. These findings present a successful strategy for broadening LFVIM applications in the regulation of ultra-low-frequency Lamb waves.

## 1. Introduction

**Figure 1:**
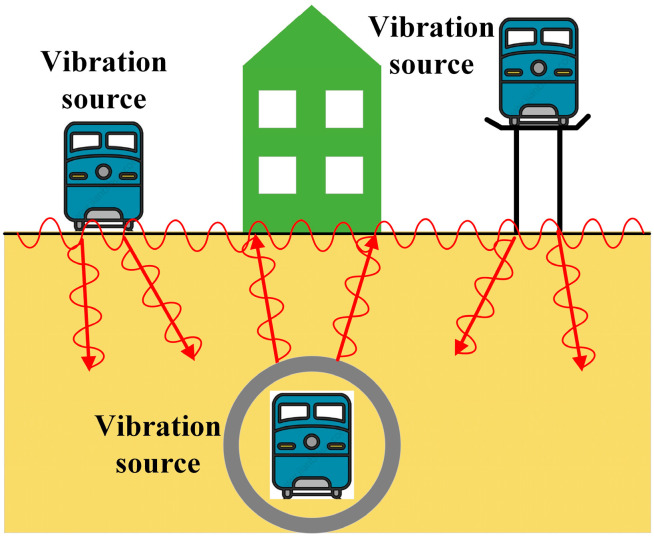
Low-frequency environmental vibration sources.

Ultra-low-frequency environmental vibrations (see Figure 1) originating from vehicles, rail transit, earthquakes and other sources have been identified as significant detrimental factors affecting structural integrity, the operation of precise instruments and residential comfort. Hayakawa et al. [1] limited the maximum frequency to 10 Hz when analyzing the geomagnetic variations associated with earthquakes. This recognition has elevated the attenuation of elastic waves to a critical priority in civil engineering. Among these, Lamb waves excited by low-frequency vibrations are a type of guided elastic wave that propagate in solid plates or layered structures with a finite thickness.

Conventional vibration isolation methods primarily focus on enhancing the overall structural stiffness or incorporating flexible elements at the foundation level [2]. The former approach often leads to excessive weight and bulk, diminishing the vibration absorption efficiency and constraining designs’ flexibility. The latter approach redirects vibration energy to designated energy-dissipative components by effectively increasing structural damping. However, effective energy absorption largely depends on the deformation of the energy-dissipative components, which may, in turn, induce elastic–plastic responses in the primary structure, potentially compromising its integrity [3,4]. Furthermore, with the increasing complexity of modern building designs, achieving effective protection against low-frequency environmental vibration through traditional vibration-resistant technologies becomes increasingly difficult. In light of these limitations, the emergence of metamaterials offers a promising way of controlling low-frequency environmental vibrations.

Metamaterials [5,6,7,8,9,10,11,12] are a kind of man-made material with elaborately designed structures that enable them to exhibit unusual and counterintuitive physical properties distinct from conventional materials, i.e., a negative Poisson’s ratio [13,14], negative refraction [15], frequency bandgaps [16], etc. For instance, Mandolesi et al. [17] explored how two dimensionless parameters affect the anisotropic elastic behavior of metamaterial unit cells, offering a strategy for tailoring specific mechanical characteristics. Liu et al. [18] studied the quasi-static compressive mechanical response of spinodoid metamaterials, using both experimental and numerical methods to characterize their macroscopic mechanical behavior. Zhang et al. [19] proposed a theoretical framework to predict the acoustic absorption performance of metamaterial absorbers with stochastic features, offering a valuable tool for understanding the influence of structural randomness on system-level acoustic behavior. Although these metamaterials exhibit exceptional mechanical properties, the integration of bandgap behavior with auxetic characteristics provides a synergistic strategy for vibration control. This combination not only enhances energy dissipation but also improves the ability to manipulate the propagation of mechanical waves, making it particularly effective for addressing low-frequency vibration challenges.

Among various metamaterials, phononic crystals (PnCs) [20,21] represent a prominent class. These materials consist of periodic structures made of multiple different types of elastic materials. These structures are designed to generate frequency bandgaps, wherein elastic waves are prohibited from propagating within specific frequency ranges, while allowing lossless propagation in other frequency bands. Based on this concept, LFVIMs using a local resonance mechanism [22] have garnered significant interest in recent years, particularly to manipulate elastic waves [23]. The local resonance concept was first proposed by Liu et al. [24], and it enables the generation of low-frequency bandgaps using small-scale structures, effectively overcoming the limitations of the Bragg scattering mechanism typically employed in PnCs. Numerous innovative LFVIM designs have emerged in recent years, with particular applications in seismic metamaterials (SMs). For instance, Du et al. [25] developed a broadband SM utilizing H-shaped fractal pillars, demonstrating its band structures for surface waves through finite element simulations. Wang et al. [26] proposed a petal-shaped SM, capable of forming ultra-low frequency bandgaps, effectively covering the frequency range with the highest concentration of Lamb wave energy. Yan et al. [4,27] proposed an SM foundation consisting of an outermost concrete layer, middle rubber layer and inner iron core, aimed at isolating Lamb waves. Vibration experiments demonstrated that significant vibration attenuation is achieved with this SM foundation when the exciting frequency applied falls within the bandgaps. Additionally, Jain et al. [28] highlighted the advantage of utilizing a one-dimensional, three-component periodic structure, which is more efficient at generating bandgaps at lower frequencies compared to two-component elements.

A significant portion of the existing research on LFVIMs focuses on designs utilizing conventional materials characterized by a positive Poisson’s ratio. Recently, auxetic metamaterials characterized by a negative Poisson’s ratio have exhibited promising bandgap properties for the attenuation of elastic waves. For example, Gao et al. [29] compared the bandgap characteristics of filled and unfilled auxetic metaconcrete structures perforated with peanut-shaped holes and found that filling the holes with soft rubber reduced the lower frequency bandgap within 50 Hz, even though the bandgap width was relatively narrow. Fei et al. [30] conducted both experimental and simulation studies to investigate the bandgap characteristics of a designed 3D auxetic metamaterial with an anti-tetrachiral structure, which successfully generated a bandgap in kHz. Ungureanu et al. [31] proposed re-entrant metamaterials buried in soil to block the propagation of seismic waves in the 1~40 Hz range. However, these existing wave-filtering applications based on auxetic metamaterials mainly involve elastic waves with a middle or high frequency. Vibration protection in a low-frequency environment, especially for seismic waves, remains an area that warrants further exploration. This is particularly critical, as the frequency range of seismic waves responsible for structural damage typically falls between 0 and 20 Hz [32,33,34]. Additionally, multiple approaches have been explored to generate low-frequency bandgaps by integrating auxetic foam with steel or concrete columns [2,35,36]; however, these approaches have not fully accounted for the influence of the real microstructure of auxetic foams. Actually, the bandgap properties of auxetic metamaterials are highly sensitive to their structural configurations. Therefore, it is crucial to further investigate the application of auxetic-based LFVIMs in vibration resistance, with a focus on novel designs that can enhance their performance in the context of the attenuation of low-frequency vibration waves.

In this work, a new 2D LFVIM is designed to create low-frequency bandgaps that encompass the primary frequency range of Lamb waves. The proposed LFVIM features a periodic unit cell composed of a square steel perforated auxetic core and a silicone rubber coating layer. The auxetic core can be filled by common engineering materials. A peanut-shaped perforation is introduced in this study to fabricate a perforated auxetic metamaterial due to its unique curved configuration, useful in reducing stress concentrations and tuning structural stiffness and strength. Recently, peanut-shaped perforation-based auxetic structures have demonstrated exceptional mechanical performance, including tunable negative Poisson’s ratio behavior, enhanced energy absorption and improved bending stiffness [37,38,39], and they have been applied to the fabrication of flexible thermoelectric elements [40], cellular concrete [41], buckling-restrained braces [42], shear wall [43], etc. In addition to their mechanical properties, exploring the dynamic response of peanut-shaped perforation-based auxetic structures in controlling low-frequency Lamb waves is interesting as well. Although Gao et al. [44] designed a composite resonator by inserting “hard” engineering materials into “soft” peanut-shaped perforation-based auxetic structures to achieve the manipulation of low-frequency elastic waves, the bandgap frequency was still within 10~30 Hz, beyond the ultra-low-frequency requirement in a low-frequency vibration environment. Thus, how to establish a feasible design based on peanut-shaped perforation-based auxetic structures to generate bandgaps below 10 Hz is the aim of the present study.

The organization of this work is as follows. Section 2 presents the structural design of the proposed LFVIM, introduces phononic crystal theory along with the related computational model for analyzing wave propagation mechanics and verifies the computational method employed through the simulation verification in the literature [26]. Section 3 provides a comparison in generating bandgaps between various core configurations. Section 4 conducts vibration tests utilizing scaled-down specimens. Section 5 reports the vibration modes of the two LFVIMs with unfilled/filled auxetic cores and details the vibration damping performance and the transmission spectrum of the LFVIMs under Lamb waves. Also, a parametric analysis is carried out to identify how the structural parameters influence the bandgap properties of the proposed LFVIM. Section 6 summarizes this work by illustrating the main findings.

## 2. Structural Design and Calculation Method

### 2.1. Structural Design

**Figure 2:**
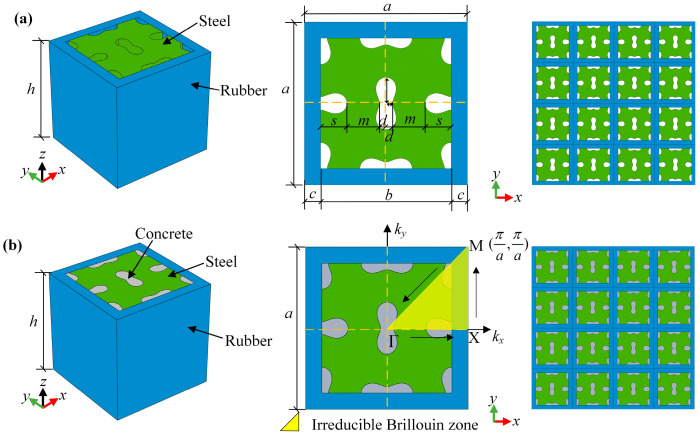
Structural designs of the present LFVIMs: (**a**) unfilled unit cell and finite array; (**b**) concrete-filled unit cell and finite array.

Figure 2a illustrates the core design of the proposed 2D LFVIM, featuring a square steel matrix perforated with orthogonally arranged peanut-shaped holes [7,29,44]. The central auxetic core is covered by a rubber coating. As depicted in Figure 2b, the 2D filled LFVIM configuration is formed by filling the peanut-shaped holes with concrete. *a* represents the lattice constant, *c* denotes the rubber layer thickness, *b* refers to the width of central steel part, *m* is the internal hole spacing determining the minimum width of the curved ligaments, and *s* and *d* represent the major and minor axes of each peanut-shaped hole, respectively. The detailed structural and physical parameters are outlined in Table 1, respectively.

### 2.2. Phononic Crystal Theory and Calculation Method

Assuming no body force and damping behavior, the dynamic response of elastic harmonic waves within the *x*-*y* plane of the phononic crystal can be described by [45]

(1) $$\nabla \cdot [C:\nabla u(r,t)]=\rho \frac{\partial^{2}u(r,t)}{\partial t^{2}}$$

where ∇ represents the Hamilton operator, **C** denotes the elastic stiffness tensor, r refers to the position vector, *t* denotes the time variable, ρ stands for the material density, and u is the displacement vector.

According to the Bloch–Floquet theorem, the displacement vector satisfies

(2) $$u(r,t)=e^{i(k\cdot r-\omega t)}\tilde{u}(r)$$

where $k=(k_{x},k_{y})$ represents the wave vector of the first Brillouin region as illustrated in Figure 2b, ω denotes the angular frequency, $\tilde{u}$ refers to the displacement modulation function, and $i=\sqrt{-1}$ is the imaginary unit.

Additionally, the structural periodicity condition requires

(3) $$\tilde{u}(r+a)=\tilde{u}(r)$$

where **a** is the lattice vector.

Substituting Equation (2) into Equation (3) yields the following equation

(4) $$u(r+a,t)=e^{i(k\cdot a)}e^{i(k\cdot r-\omega t)}\tilde{u}(r)$$

Applying finite element discretization to the elastic dynamic system consisting of Equations (1) and (4) produces the following eigenvalue equations

(5) $$[K(k)-\omega^{2}M]U=0$$

where U represents the nodal displacement vector, and K and M denote the stiffness and mass matrices, respectively.

In this study, finite element computation is implemented using COMSOL Multiphysics 6.1 software, due to its high flexibility in addressing multi-material systems, such as the present LFVIMs.

### 2.3. Mesh Convergence of FEM

The computational accuracy of the finite element method (FEM) is closely related to the mesh resolution, specifically the number of elements or nodes utilized. Hence, the FEM’s effectiveness is firstly illustrated through a mesh convergence analysis. The unfilled LFVIM with dimensions (*a*, *s*, *d*) = (1000, 160, 40) mm is chosen for the mesh convergence analysis. All the vertical edges of the unfilled LFVIM are subject to Bloch–Floquet periodic boundary conditions, which are employed in the simulation of periodic structures to model wave propagation through an infinite array by analyzing only a single unit cell. The top and bottom surfaces remain free. The wave vector k is swept along the boundary Γ−X−M−Γ of the first Brillion zone, which is the primitive cell in the reciprocal lattice and which encompasses all the unique wave vectors that characterize wave propagation in a periodic structure, according to Bloch’s theorem. This zone serves as the fundamental domain for computing the band structure. Free tetrahedral elements are used, and the number of elements ranges from 13,052 to 213,600, representing various mesh densities. These correspond to meshing strategies that transition from extra-coarse to finer settings in COMSOL.

**Figure 3:**
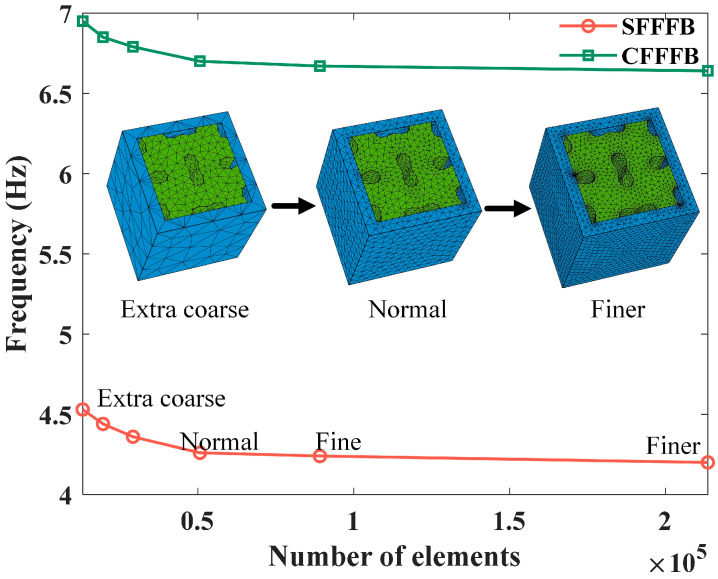
Analysis of mesh convergence of FEM for modeling the present LFVIM.

As shown in Figure 3, as the number of elements increase, both the starting frequency (SFFFB) and the cutoff frequency of the first full bandgap (CFFFB) gradually decrease, ultimately reaching convergent values. Notably, convergence is reached for both the SFFFB and CFFFB when the number of elements exceeds 50,663, which corresponds to the normal meshing strategy. Therefore, to strike an optimal balance between numerical accuracy and computational efficiency for practical simulations involving various structural configurations, the normal element-size control strategy will be adopted in subsequent simulations.

### 2.4. Method Verification

**Figure 4:**
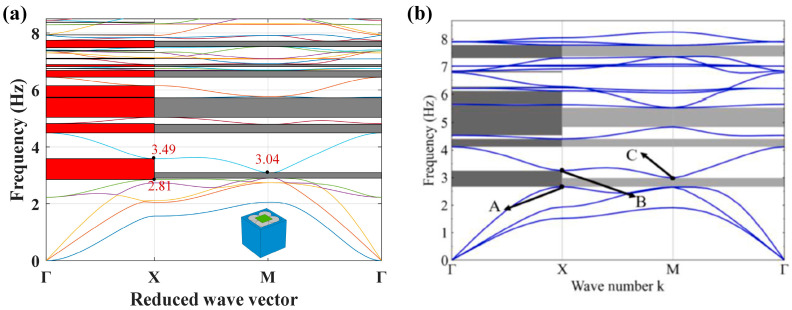
Band structures obtained through finite element method for the petal-shaped SM established in the literature [26]: (**a**) Result of verification; (**b**) Result of literature [26].

To validate the reliability of the computational approach employed in this work, the petal-shaped SM presented in the literature [26] is analyzed using COMSOL Multiphysics software. The applied boundary conditions and the wave vector scanning path remain identical to those described in the previous section. The structural and physical parameters of the simulated model are consistent with those presented in the literature [26]. Figure 4 presents the simulated results, revealing that the first *x*-direction bandgap extends from 2.81 Hz to 3.49 Hz, while the first full bandgap occurs within a narrower range of 2.81 Hz to 3.04 Hz. The simulated results exhibit excellent agreement with those reported in [26], with only minor discrepancies observed. These slight differences may be attributed to the variations in element type and meshing strategy used in the simulations. Therefore, the FEM used in this study for analyzing the SM has been validated as accurate for such analyses. Additionally, it is observed that the petal-shaped SM established in [26] can generate multiple low-frequency bandgaps below 10 Hz, although the relative width of these bandgaps is relatively narrow. This observation motivates the design of a new LFVIM aimed at expanding the low-frequency bandgap range.

## 3. Comparison of Bandgap Characteristics for Various Core Configurations

Although the mechanical performance of auxetic structures with peanut-shaped perforations has been explored recently, their potential bandgap characteristics remain unexplored and have not been systematically compared with those of other core configurations. To address this gap, the band structures of the unfilled structures with varying core configurations are comparatively analyzed, involving peanut-shaped, rectangular and elliptical holes. All three types of perforated cores are analyzed at two distinct porosity levels: low porosity (approximately 12%) and high porosity (approximately 34%).

**Figure 5:**
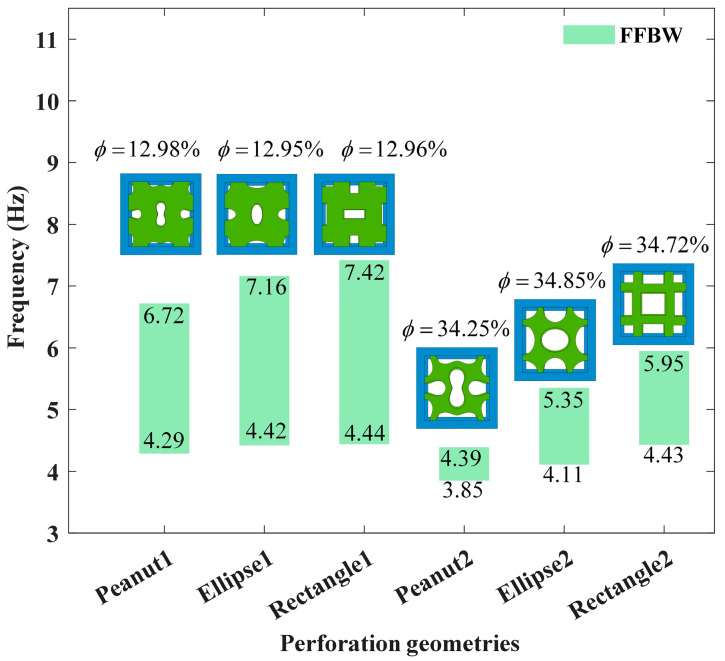
Comparison of bandgap characteristics for various core configurations.

As illustrated in Figure 5, the perforated cores can achieve tunable bandgaps by adjusting the porosity. As the porosity increases, the bandgap moves downward, exhibiting great potential in manipulating Lamb waves in the low-frequency regime. Notably, the peanut-shaped perforations exhibit the lowest starting frequency compared to the rectangular and elliptical perforations. Therefore, in the following analysis, only the peanut-shaped perforations are investigated.

## 4. Vibration Experiment

**Figure 6:**
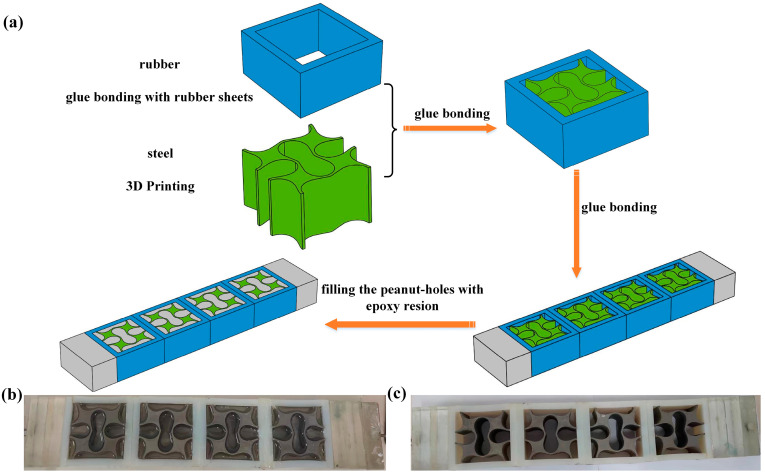
Preparation process: (**a**) Manufacturing of the specimen. (**b**) Specimen of the filled LFVIM. (**c**) Specimen of the unfilled LFVIM.

Limited by the excitation generator’s power, the vibration experiments are conducted using two scaled-down specimens, each comprising four unit cells, as depicted in Figure 6. These experiments aim to demonstrate the low-frequency damping behavior of the presented LFVIMs while also validating the FEM used in this study. The parameters of the unit cell for the specimens are presented in Table 2, while the density and elastic modulus of the materials are calibrated in Appendix A. Due to the size limitation of the specimens, it is not feasible to fill the perforations with concrete. To address this, a highly transparent epoxy resin with a good stability and low shrinkage is used as the filler, because its band structures are similar to that of concrete filler. The polyurethane glue is employed to bond the components within each unit cell as well as between the individual unit cells. Additionally, due to the high elasticity of rubber, achieving a uniform acceleration input and output is challenging. To overcome this, epoxy resin plates are affixed to both ends of the specimens to ensure uniform acceleration at both the input and output ends. The damping coefficients for rubber, epoxy resin and steel are applied separately in the simulation calculation, with values of 0.01, 0.005 and 0.0001, respectively [26].

**Figure 7:**
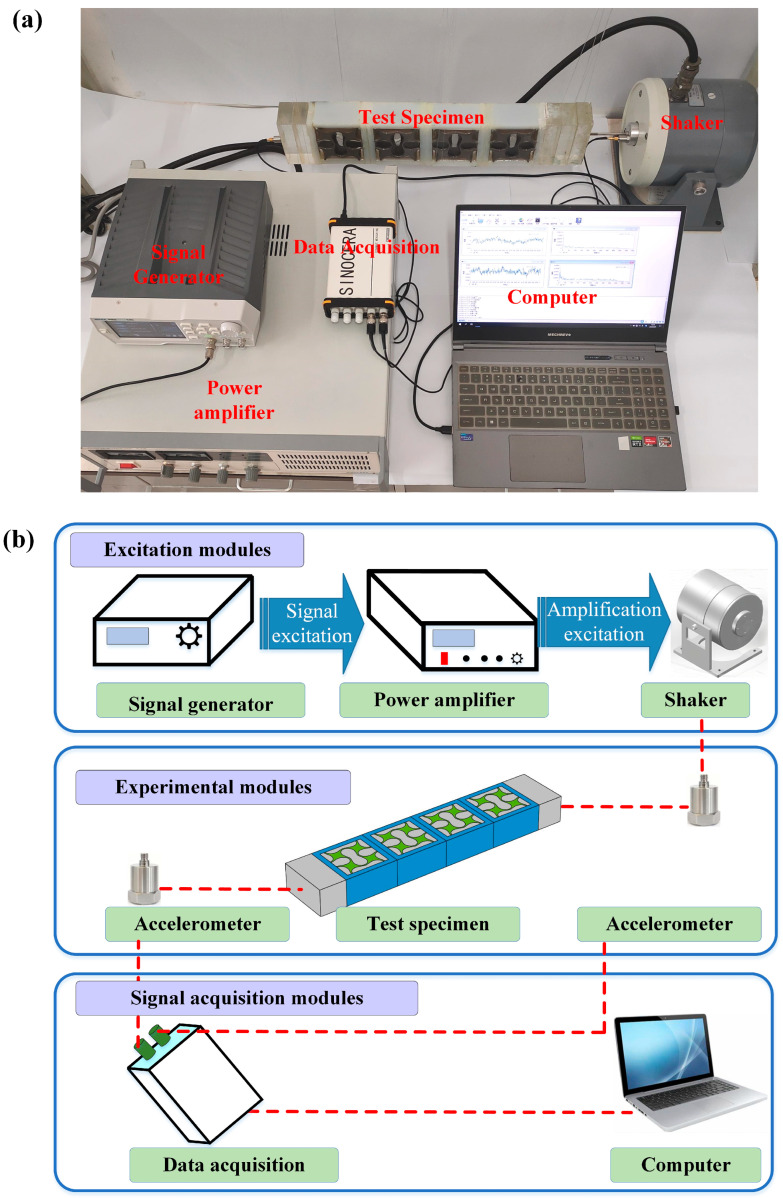
Vibration experiment setup for the scaled-down specimens: (**a**) Experimental instruments. (**b**) Schematic diagram.

Figure 7 illustrates the experimental system, which is composed of the excitation, experimental and signal acquisition modules. The excitation module includes a signal generator, a power amplifier and a shaker. The experimental module comprises the test specimen along with the acceleration sensors for response measurement. The signal acquisition module consists of a data acquisition device and a computer. The shaker is positioned horizontally at the input end of the specimen, while the other end of the specimen serves as the output end. Two acceleration sensors are respectively attached to the input and output ends to measure acceleration during the application of simple harmonic excitation generated by the signal generator. The acquired acceleration data is subsequently analyzed using the control software of the YE7600 data acquisition device.

**Figure 8:**
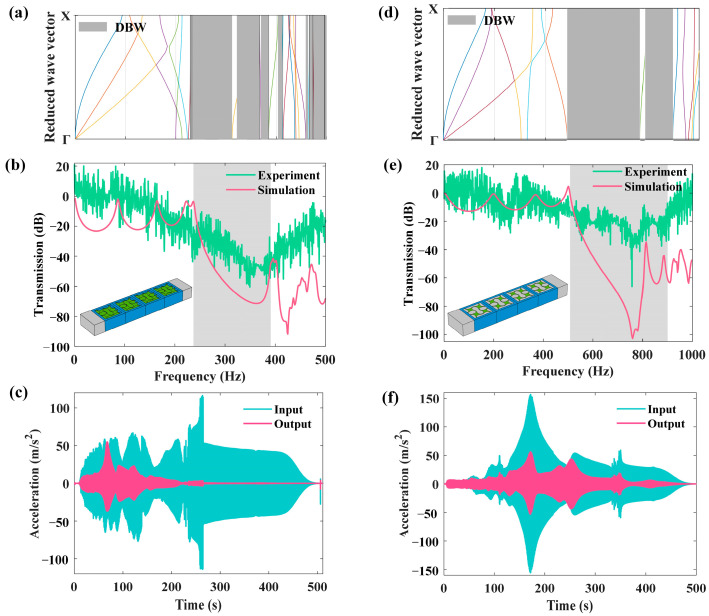
The dispersion curves, the transmission spectra and the acceleration histories shown in (**a**–**c**) and (**d**–**f**) for the unfilled and filled LFVIM specimens obtained from the simulation and experiment.

According to the Γ−X directional bandgap width (DBW) of both the unfilled and filled LFVIMs, as depicted in Figure 8a,d, the signal generator emits frequency sweep signals ranging from 1 to 500 Hz for the unfilled LFVIM and from 1 to 1000 Hz for the filled LFVIM. The scanning time is set to 500 s, corresponding to the maximum sweep time of the signal generator used in this experimental system. Figure 8b,e present a comparison between experimental and simulated transmission spectra for the unfilled LFVIM and filled LFVIM, respectively. The experimental curves follow a pattern similar to the simulated values. Specifically, the experimental data reveals that the unfilled LFVIM begins to exhibit vibration isolation after 190 Hz, with the maximum attenuation reaching 60 dB. In contrast, the filled LFVIM shows an attenuation starting at 430 Hz, with an amplitude of 66 dB. Moreover, compared to the experimental results, the starting attenuation frequency of the two specimens from the simulation is slightly higher than that from the experiment. This difference can be attributed to imperfections at the interface between the rubber and the steel during the vibration process, where the presence of gaps weakens the transmission of acceleration. This effect also is reflected in the reduced attenuation amplitude observed in the experimental results. The experimental attenuation in the bandgap frequencies is smaller than that predicted by the simulation. Additionally, the damping of the silicone rubber decreases with increasing loading frequency, while the rubber’s elastic modulus rises due to repeated extrusion deformation of the rubber layer throughout the experiment [26]. Nevertheless, the rubber’s elastic modulus remains constant in the simulation, leading to the observed differences between the experimental and simulation results. Figure 8c,f present the acceleration histories of the unfilled and filled LFVIM structures, respectively. It is indicated that the acceleration at the output end of the unfilled LFVIM tends to be stable and is significantly smaller than that at the input end after 230 s, while the acceleration at the output end of the filled LFVIM becomes apparent after 250 s.

## 5. Results and Discussion

The preceding analysis demonstrates that this present LFVIM design effectively modulates Lamb wave propagation. Simultaneously, the established FEM model exhibits a reliable reproducibility of results from the experiment and available literature. Therefore, the validated finite element model is subsequently employed to conduct an in-depth investigation into the bandgap performance of the present LFVIM to identify the role of structural parameters.

### 5.1. Band Curve Analysis

**Figure 9:**
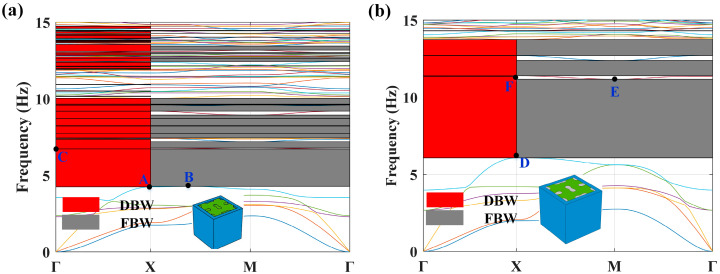
Band structures: (**a**) The unfilled LFVIM, where points A, B and C correspond to the starting and cutoff points of the first directional/full bandgap, respectively. (**b**) The filled LFVIM, where points D, E and F are the starting and cutoff points of the first directional/full bandgap, respectively.

The dispersion curves for the two LFVIMs with parameters (*a*, *s*, *d*) = (1000, 160, 40) mm are presented in Figure 9. To provide a clear representation for the bandgap properties, the relative bandgap width (RBW) is defined as [46]

(6) $$\Psi =\frac{2(f_{ub}-f_{lb})}{\left(f_{ub}+f_{lb}\right)}\times 100\%$$

where $f_{lb}$ and $f_{ub}$ denote the lower and upper bounds of each bandgap, respectively.

Firstly, as indicated by the gray area of Figure 9a, the unfilled LFVIM generates multiple full bandgaps within 15 Hz, resulting in a total width of 5.74 Hz. The first full bandgap in [4.29, 6.72] Hz with a relative width of 44.25% is the main bandgap. Simultaneously, the directional bandgaps along the Γ−X direction are highlighted in the red shaded region of Figure 9a. The total directional bandgap width is 7.49 Hz, and the frequency range of the first directional bandgap is [4.09, 6.72] Hz. Compared to the unfilled LFVIM, the introduction of concrete in the filled LFVIM alters the band structures, as depicted in Figure 9b. The filled LFVIM generates three full bandgaps within 15 Hz, with a total width of 7.09 Hz. The first full bandgap lies in the range of 6.08–11.16 Hz, with a relative width of 58.93%. Moreover, the total directional bandgap width of the filled LFVIM is 8.11 Hz, with the first directional bandgap spanning from 6.08 Hz to 11.36 Hz. These results manifest that although the first full bandgap of the filled LFVIM starts at a relatively higher frequency compared to the unfilled LFVIM, the bandgap width is more than double that of the unfilled LFVIM.

**Figure 10:**
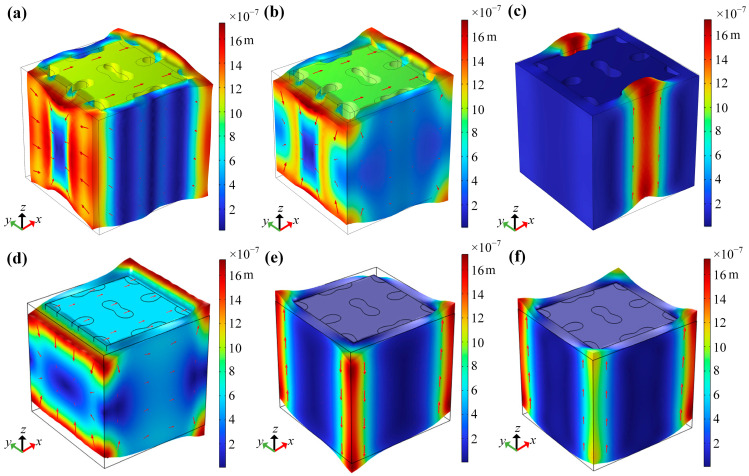
The vibration pattern of the present LFVIMs at start and cutoff frequencies: (**a**–**c**) Vibration patterns of the unfilled LFVIM at points A, B and C. (**d**–**f**) Vibration patterns of the filled LFVIM at points D, E and F.

Additionally, for the unfilled LFVIM, the starting frequencies of the first directional and complete bandgaps are marked by point A at 4.09 Hz and point B at 4.29 Hz, respectively. However, their cutoff points keep the same frequency of 6.72 Hz (point C). Figure 10a–c illustrate the vibration modes at points A, B and C, respectively. Figure 10a,b show that the vibration modes at point A and B are similar. The internal steel core translates along the *x*-direction, driving the silicone rubber layer to deform perpendicular to the *x*-direction. This deformation causes inward motion on the left side of the rubber layer (in the *y*-*z* plane) and outward deformation on the right side, generating a resonance that leads to the formation of the bandgap. The vibration patterns at the cutoff point C (Figure 10c) are predominantly concentrated in the upward deformation of the middle part of the rubber covering layer on the front and back sides, whilst the movement of the internal steel core ceases. The release of the coupling effect results in the disappearance of the bandgap.

For the filled LFVIM, the directional and full bandgaps start at the same frequency of 6.08 Hz (point D). Nevertheless, the cutoff frequencies for these two bandgaps differ. The full bandgap terminates at 11.16 Hz (point E), while the directional bandgap ends at 11.36 Hz (point F). Figure 10d–f display the corresponding vibration patterns at points D, E and F. Figure 10d indicates that the vibration pattern at the starting point D closely resembles those at points A and B. The vibration pattern at point E, shown in Figure 10e, is primarily localized to the twisting deformation of the four rubber faces, whilst the internal core remains stationary. Similarly, at the cutoff point F for the directional bandgap (Figure 10f), the internal core keeps stationary, while the vibration is primarily concentrated on the upward motion of four corners of the rubber layer in the *z*-direction.

### 5.2. Transmission Response Analysis

**Figure 11:**
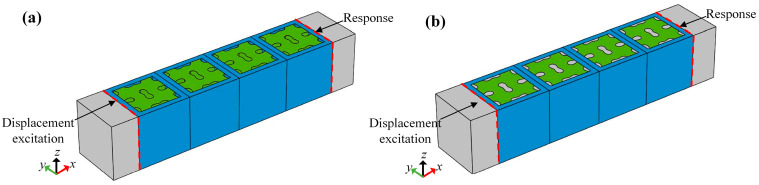
Frequency response analysis: (**a**) Unfilled array. (**b**) Filled array.

**Figure 12:**
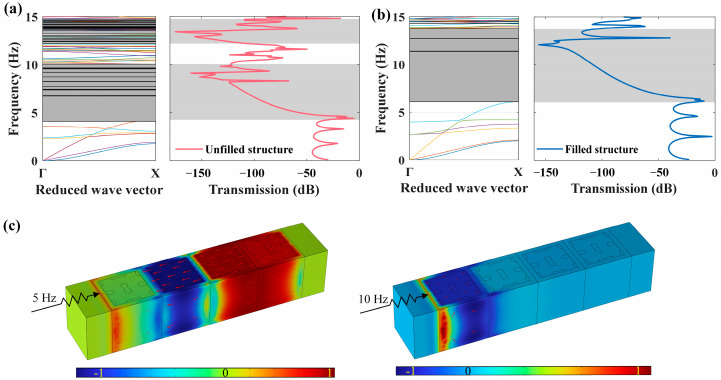
The transmission loss characteristic for the proposed LFVIMs. (**a**,**b**) Transmission loss spectra of the unfilled/filled LFVIM in the *x*-direction. (**c**) Vibration response of the filled LFVIM for the incident waves at frequencies of 5 Hz (beyond the bandgap) and 10 Hz (within the bandgap).

In the preceding section, the dispersion curves for a single unit cell are calculated to identify the locations of the bandgaps. To further validate the existence of bandgaps, a finite structure comprising four unit cells is constructed to calculate the transmission loss spectrum, as illustrated in Figure 11. Concrete blocks are placed on both the left and right ends, serving as perfectly matched layers (PMLs) to minimize the Lamb wave reflection from the sides. The Bloch–Floquet periodicity boundary conditions are enforced in the *y*-direction. A defined displacement excitation $U_{exc}$ is imposed on the left end of the finite structure, while the displacement response $U_{res}$ is measured on the right end. The transmission coefficient of the finite structure is determined using the following formula [29]:

(7) $$\mathrm{Transmission}=20\log_{10}\frac{U_{res}}{U_{exc}}$$

where a positive transmission coefficient indicates an amplified excitation, while a negative coefficient signifies an attenuated excitation. Figure 12a,b display the transmission loss spectra corresponding to the unfilled and filled finite structures, respectively, indicating a strong consistency with the dispersion curves along the *x*-direction. Both of the two LFVIMs display significant attenuation, with maximum values exceeding 160 dB, signifying substantial shock absorption. Furthermore, the same conclusion can be drawn that although the attenuation in the filled LFVIM begins at a higher frequency compared to the unfilled LFVIM, the frequency range over which the attenuation occurs is greatly widened. Thus, the filled LFVIM structure is further investigated in the subsequent discussion.

In addition, the wave-filtering performance of the filled LFVIMs is assessed, and the displacement amplitude fields for specific incident wave frequencies are presented in Figure 12c. It is shown that when the frequency of an incident elastic wave is beyond the bandgap (e.g., 5 Hz), it is able to propagate through the finite array, inducing a strong vibration throughout the whole array. Oppositely, if the frequency lies within the bandgap (e.g., 10 Hz), an effective propagation block is observed, with no displacement detected on the right end of the finite structure, confirming the filtering capability of the LFVIM.

### 5.3. Effects of Influencing Factors

This section explores how geometric and physical parameters impact the bandgap characteristics of the filled LFVIM, aiming to identify optimal structures that yield the most favorable bandgap properties.

#### 5.3.1. Effect of Auxetic Core’s Porosity

The porosity is a key geometrical parameter related to the size of perforations. In order to investigate its impact on bandgap characteristics, four distinct configurations of the peanut-shaped hole are generated by adjusting the shape coefficient φ=s/d to 2.5, 3, 3.5 and 4. For each configuration, the semi-width d varies from 40 mm to 70 mm, and the corresponding semi-length s is adjusted accordingly, thereby altering the porosity of the auxetic steel core.

**Figure 13:**
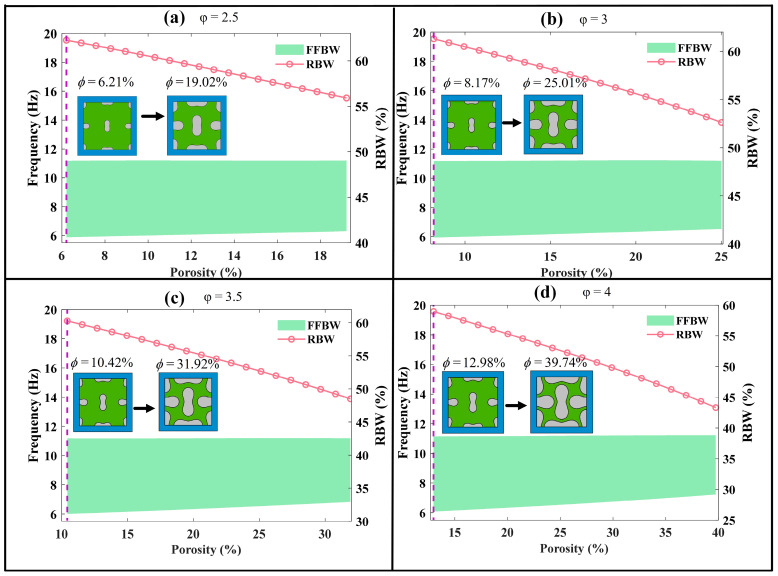
Impact of porosity on the bandgap characteristics of the filled LFVIM: (**a**) φ=2.5; (**b**) φ=3; (**c**) φ=3.5; (**d**) φ=4. The schematic diagram represents the plan view.

Figure 13 illustrates the relationship between the porosity and bandgap characteristics. It is evident that the porosity of auxetic core affects the starting frequency, cutoff frequency and the first full bandgap width. Specifically, the curve of SFFFB gradually rises with the increase in porosity. This trend can be attributed to the reduction in mass of the internal core as the porosity increases, leading to a shift of the starting frequency to higher values. Conversely, the curve of CFFFB remains relatively unaffected by changes in porosity. This can be explained by the vibration mode observed at the cutoff frequency primarily occurring in the silicone rubber layer rather than in the internal core, as depicted in Figure 10e.

Consequently, the FFBW progressively decreases with increasing porosity. The maximum bandgap widths are 5.31 Hz, 5.25 Hz, 5.18 Hz and 5.08 Hz, while the corresponding relative bandgap widths are 62.27%, 61.31%, 60.27% and 58.99% for the cases of φ= 2.5, 3, 3.5 and 4, respectively.

#### 5.3.2. Effect of Coating Material

As well as the geometric factors, the physical properties of each component material can significantly influence the bandgap properties of the LFVIM. To investigate the impact of material properties, the geometric parameters are kept invariant, as specified in Table 1.

**Figure 14:**
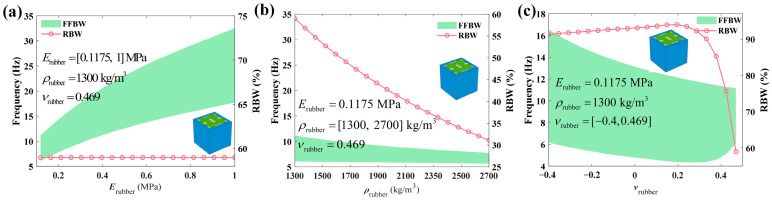
Influences of the physical properties of the coating layer on the bandgap: (**a**) $E_{\mathrm{rubber}}$; (**b**) $\rho_{\mathrm{rubber}}$; (**c**) $\nu_{\mathrm{rubber}}$.

Figure 14a illustrates how the bandgap frequencies change with the varying of the rubber’s elastic modulus. As the elastic modulus increases from 0.1175 MPa to 1 MPa, both the SFFFB and CFFFB move towards a higher frequency. Additionally, the overall bandgap broadens as well. This behavior can be attributed to the increased stiffness of the silicone rubber, which causes the bandgap to shift to higher frequencies, assuming the material density remains constant. Notably, Figure 14a depicts that although the rubber’s elastic modulus increases, the RBW keeps almost invariant at 58.99%, indicating that variations in the rubber’s elastic modulus have a minimal impact on the RBW. Figure 14b manifests how the bandgap changes as the rubber’s density ranges from 1300 kg/m^3^ to 2700 kg/m^3^. It is evident that the SFFFB decreases slightly with the rise in rubber density, whilst the CFFFB reduces rapidly. As the rubber stiffness keeps invariable, the rise in rubber density leads to a higher mass, which reduces the structural characteristic frequency and consequently shifts the bandgap to lower frequency. Figure 14c describes the variations in bandgaps as the Poisson’s ratio of coating material increases from −0.4 to 0.469. Here, the negative Poisson’s ratio means that the coating is auxetic foam [2,35,36]. It is indicated that the CFFFB basically shifts towards lower frequencies with an increasing Poisson’s ratio, whereas the SFFFB decreases first and then rises. This causes the RBW to initially increase, reaching a peak value of 93.95% when the Poisson’s ratio is 0.2, and then dramatically decrease to a minimum value of 58.99%. This trend can be attributed to the regulatory effect of variations in Poisson’s ratio on the coupling effect between the volumetric deformation and shear response of the material. Specifically, within the negative Poisson’s ratio range, the material’s lateral expansion effect is enhanced, which strengthens the coupling of local vibration modes and further promotes the optimization of bandgap characteristics.

#### 5.3.3. Effect of Core Material

**Figure 15:**
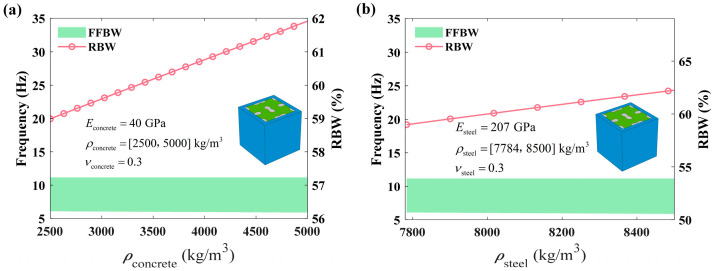
Effect of the density of the auxetic core on the bandgap: (**a**) filling material; (**b**) base material.

The influence of the internal core on bandgap characteristics is investigated. As shown in Figure 15, the CFFFB remains unchanged at 11.16 Hz, which corresponds to the uncoupled vibration mode depicted in Figure 10. In contrast, the SFFFB slightly descends as the density of both concrete and steel increases.

**Figure 16:**
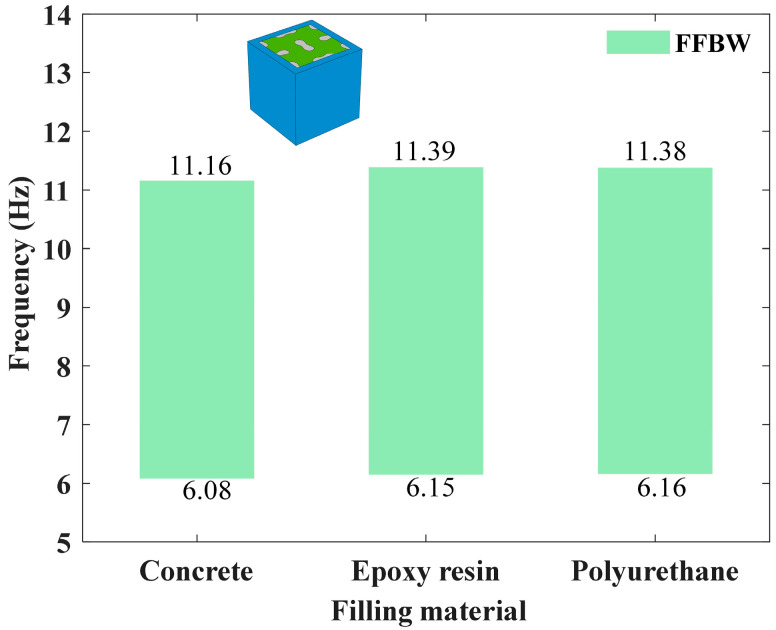
Effect of filling material.

Finally, the influence of three typical filling materials on the bandgap characteristics of the filled LFVIM is investigated, with geometric parameters fixed at (*a*, *s*, *d*) = (1000, 160, 40) mm. The base material remains steel, while the filling materials considered include concrete, epoxy resin and polyurethane. As shown in Figure 16, the three filling materials exhibit comparable bandgap widths and frequency ranges, primarily attributed to the vibration modes at the starting and cutoff frequencies illustrated in Figure 10d,e.

## 6. Conclusions

In this study, a novel LFVIM composed of a silicone rubber coating and an auxetic steel core perforated with peanut-shaped holes is proposed, aimed at achieving an ultra-wide bandgap over the low-frequency range to attenuate low-frequency Lamb waves. Additionally, a concrete-filled LFVIM structure is created for a comparative study. Both unfilled and concrete-filled LFVIMs are examined to explore their bandgap performance. Numerical simulations and scaled vibration experiments confirm the LFVIMs’ ability to generate ultra-wide, low-frequency bandgaps for the effective attenuation of Lamb waves. The concrete-filled LFVIM exhibits an enhanced bandgap performance by merging multiple narrow bandgaps into broader ones within the 15 Hz range, with the first full bandgap expanding by 14.68% compared to the unfilled structure, specifically from [4.29, 6.72] Hz to [6.08, 11.16] Hz. Parametric analyses indicate that increasing the porosity of the auxetic core raises the starting frequency but narrows the bandgap. Additionally, the coating properties, especially elastic modulus and Poisson’s ratio, significantly affect the relative RBW. A peak RBW of 93.95% is achieved at a Poisson’s ratio of 0.2.

In summary, this work presents a novel design strategy for an LFVIM to effectively attenuate Lamb waves within the low-frequency range. The investigation is beneficial for broadening the potential applications of LFVIMs in low-frequency vibration environments and related fields. However, as the present work mainly focuses on the attenuation of low-frequency Lamb waves, the attenuation of low-frequency surface waves, which are known to cause considerable damage to building structures, will be investigated in future work.

## Figures and Tables

**Table 1:** Structural and physical parameters of the unit cell [35].

a (mm)	c(mm)	b(mm)	h (mm)	d(mm)	s(mm)	m (mm)
1000	100	800	1000	40	160	200
Material	E(MPa)		ν	$\rho (kg/m^{3})$		
Concrete	4 × 10^4^		0.300	2500		
Rubber	0.1175		0.469	1300		
Steel	2.07 × 10^5^		0.300	7784		

**Table 2:** Geometric parameters of test specimen.

a (mm)	c(mm)	b(mm)	h (mm)	d(mm)	s(mm)
100	10	80	50	7.5	30
Material	E(MPa)		ν	$\rho (kg/m^{3})$	
Epoxy resin	1620		0.300	1835	
Rubber	4.28		0.469	1185	
Steel	2.07 × 10^5^		0.300	7850	

**Figure A1:**
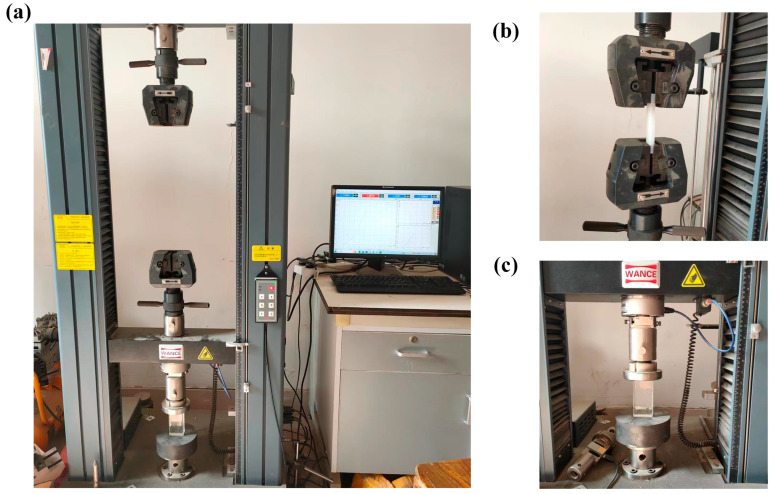
Experimental procedure: (**a**) WANCE electrohydraulic universal machine (ETM504C). (**b**) Quasi-static uniaxial compression test of rubber block. (**c**) Quasi-static uniaxial compression test of epoxy resin block.

**Figure A2:**
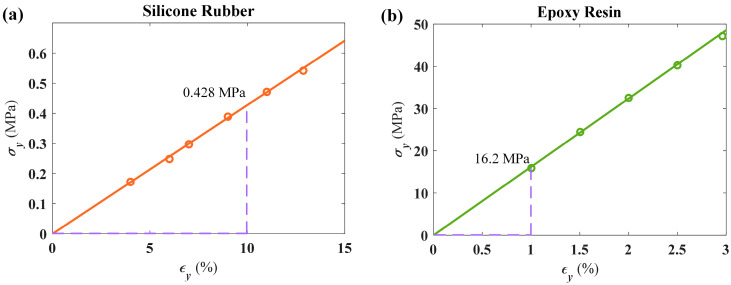
Stress–strain relationship: (**a**) Silicone rubber. (**b**) Epoxy resin.

## Data Availability

The original contributions presented in this study are included in the article. Further inquiries can be directed to the corresponding author.

## Appendix A. Calibration of Material Properties

In this work, the material properties of rubber and epoxy resin are determined through experimental tests. Specifically, the quasi-static uniaxial tensile test is conducted for rubber, while the quasi-static uniaxial compression test is carried out for epoxy resin, as seen in Figure A1. The size of the rubber specimen in Figure A1b is 20×100×10 mm, and the dimension of the epoxy resin block in Figure A1c is 50×100×25 mm. The corresponding cross-sectional areas of the rubber block and epoxy resin block along the thickness direction are 200 ${\mathrm{mm}}^{2}$ and 1250 ${\mathrm{mm}}^{2}$, respectively. The real-time force applied to the specimen in the *y*-direction can be obtained through the built-in force sensor, and the displacement can be measured by a displacement gauge. Then the stress–strain relationships of the rubber and epoxy resin can be obtained, as shown in Figure A2. The experimental data are fitted linearly. Then the slopes corresponding to the fitted lines are the equivalent elastic moduli of the rubber and epoxy resin, which are 4.28 MPa and 1.62 GPa, respectively.

## References

[1] Hayakawa M., Hattori K., Ohta K. (2007). Monitoring of ULF (Ultra-Low-Frequency) Geomagnetic Variations Associated with Earthquakes. Sensors.

[2] Luo Y.M., He C., Tao Z., Hao J., Xu H.H., Zhang Y., Zhang F., Ren X. (2024). A surface-wave seismic metamaterial filled with auxetic foam. Int. J. Mech. Sci..

[3] Kiggins S., Uang C.M. (2006). Reducing residual drift of buckling-restrained braced frames as a dual system. Eng. Struct..

[4] Yan Y., Laskar A., Cheng Z., Menq F., Tang Y., Mo Y.L., Shi Z. (2014). Seismic isolation of two dimensional periodic foundations. J. Appl. Phys..

[5] Ren X., Das R., Tran P., Ngo T., Xie Y. (2018). Auxetic metamaterials and structures: A review. Smart Mater. Struct..

[6] Kolken H.M.A., Zadpoor A.A. (2017). Auxetic mechanical metamaterials. RSC Adv..

[7] Wang H., Xiao S.H., Wang J.S. (2021). Disordered auxetic metamaterials architected by random peanut-shaped perturbations. Mater. Des..

[8] Liu Y.X., Dou S.H., Du Y.P., Liang R.Z., Yue S.Y., Zhao L.X., Liu F., Sun Z.Y., Yang J. (2025). Enhanced broadband low-frequency performance of negative Poisson’s ratio metamaterials with added mass. Sci. Rep..

[9] Yang F., Fan Y.L., Yang J.S., Wu Y., Dai G.H. (2025). A new cylindrical acoustic metamaterial for low-frequency vibration attenuation. Structures.

[10] Han S.H., Han Q., Ma N.F., Li C.L. (2023). Design and reinforcement-learning optimization of re-entrant cellular metamaterials. Thin-Walled Struct..

[11] Zhang Q., Sun Y.X. (2024). Low frequency bandgap and high stiffness of innovative auxetic metamaterial with negative thermal expansion. Thin-Walled Struct..

[12] Lymperopoulos P.N., Theotokoglou E.E. (2025). Numerical analyses of pentamodes metamaterials behavior under harmonic loading conditions. Eur. J. Mech. A/Solids.

[13] Alderson K.L., Pickles A.P., Neale P.J., Evans K.E. (1994). Auxetic polyethylene: The effect of a negative poisson’s ratio on hardness. Acta Metall. Mater..

[14] Lakes R. (1987). Foam Structures with a Negative Poisson’s Ratio. Science.

[15] Liu J., Hou Z.L., Fu X.J. (2015). Negative refraction realized by band folding effect in resonator-based acoustic metamaterials. Phys. Lett. A.

[16] Jia G.F., Shi Z.F. (2010). A new seismic isolation system and its feasibility study. Earthq. Eng. Eng. Vib..

[17] Mandolesi B., Iandiorio C., Belardi V.G., Vivio F. (2025). Spinodal decomposition-inspired metamaterial: Tailored homogenized elastic properties via the dimensionless Cahn-Hilliard equation. Eur. J. Mech. A/Solids.

[18] Liu Y.J., Wang H.Y., Yan L.W., Huang J.Z., Liang Y.J. (2024). Mechanical properties of homogeneous and functionally graded spinodal structures. Int. J. Mech. Sci..

[19] Zhang W.Z., Zhao Y.H. (2025). Sound absorption characteristics of the metamaterial with stochastic parameters. Int. J. Mech. Sci..

[20] Chen Y.F., Guo D., Li Y.F., Li G.Y., Huang X.D. (2019). Maximizing wave attenuation in viscoelastic phononic crystals by topology optimization. Ultrasonics.

[21] Hsu J.C., Wu T.T. (2007). Lamb waves in binary locally resonant phononic plates with two-dimensional lattices. Appl. Phys. Lett..

[22] Chu J.M., Zhou G.J., Liang X., Liang H.F., Yang Z., Chen T. (2023). A metamaterial for low-frequency vibration damping. Mater. Today Commun..

[23] Achaoui Y., Antonakakis T., Brûlé S., Craster R.V., Enoch S., Guenneau S. (2017). Clamped seismic metamaterials: Ultra-low frequency stop bands. New J. Phys..

[24] Liu Z.Y., Zhang X.X., Mao Y.W., Zhu Y.Y., Yang Z.Y., Chan C.T., Sheng P. (2000). Locally Resonant Sonic Materials. Science.

[25] Du Q.J., Zeng Y., Xu Y., Yang H.W., Zeng Z.X. (2018). H-fractal seismic metamaterial with broadband low-frequency bandgaps. J. Phys. D Appl. Phys..

[26] Wang Y., Yang F., Yang J.S., Tong L.L., Li S., Liu Q., Hou G.L., Sun P.D., Xing M., Zheng G. (2023). Study on vibration damping performance of a petal-shaped seismic metamaterial. Structures.

[27] Yan Y., Cheng Z., Menq F., Mo Y.L., Tang Y., Shi Z. (2015). Three dimensional periodic foundations for base seismic isolation. Smart Mater. Struct..

[28] Jain S., Pujari S., Laskar A. (2021). Investigation of one dimensional multi-layer periodic unit cell for structural base isolation. Structures.

[29] Gao Y.T., Wang H. (2024). Comparative investigation of full bandgap behaviors of perforated auxetic metaconcretes with or without soft filler. Mater. Today Commun..

[30] Fei X., Jin L., Zhang X.J., Li X., Lu M.H. (2020). Three-dimensional anti-chiral auxetic metamaterial with tunable phononic bandgap. Appl. Phys. Lett..

[31] Ungureanu B., Achaoui Y., Enoch S., Brûlé S., Guenneau S. (2015). Auxetic-like metamaterials as novel earthquake protections. EPJ Appl. Metamater..

[32] Martelli A., Forni M. (2010). Seismic isolation and other antiseismic systems: Recent applications in Italy and worldwide. Seism. Isol. Prot. Syst..

[33] Zeng Y., Peng P., Du Q.J., Wang Y.S., Assouar B. (2020). Subwavelength seismic metamaterial with an ultra-low frequency bandgap. J. Appl. Phys..

[34] Zeng Y., Xu Y., Deng K.K., Zeng Z.X., Yang H.W., Muzamil M., Du Q.J. (2018). Low-frequency broadband seismic metamaterial using I-shaped pillars in a half-space. J. Appl. Phys..

[35] Huang T.T., Ren X., Zeng Y., Zhang Y., Luo C., Zhang X.Y., Xie Y.M. (2021). Based on auxetic foam: A novel type of seismic metamaterial for Lamb waves. Eng. Struct..

[36] Li P.F., Yang F., Zhao M., Du Z.L., Fan H.L. (2024). A new seismic metamaterial design with ultra-wide low-frequency wave suppression band utilizing negative Poisson’s ratio material. Eng. Struct..

[37] Zhang C., Xiao S.H., Qin Q.H., Wang H. (2021). Tunable compressive properties of a novel auxetic tubular material with low stress level. Thin-Walled Struct..

[38] Zhang Z., Lei Y., Wang H. (2025). Deformation and energy absorption characteristics of graded auxetic metamaterials featuring peanut-shaped perforations under in-plane compression. Int. J. Solids Struct..

[39] Gong Q., Wang D., Dong Q., Wang H. (2025). Bending resistance and transverse energy absorption behaviors of auxetic tubes with orthogonal pattern of peanut-shaped perforations. Mech. Adv. Mater. Struct..

[40] Choi H., Min B.-K., Joo S.-J., Kim B.-S., Lee K., Kang H., Sim Y.H., Yun M.J., Lee D.Y., Cha S.I. (2023). Partially Air-Filled Skin-Attachable Deformable Gasket with Negative Poisson’s Ratio for Highly-Efficient Stretchable Thermoelectric Generators. Adv. Energy Mater..

[41] Xie J., Xu Y., Meng Z., Liang M., Wan Z., Šavija B. (2024). Peanut shaped auxetic cementitious cellular composite (ACCC). Constr. Build. Mater..

[42] Zhu Y., Wang J., Cai X., Xu Z., Wen Y. (2023). Cyclic behavior of ellipse and peanut-shaped perforated buckling-restrained braces. Eng. Struct..

[43] Wang J., Zhu Y., Cai X., Wen Y., Wang P. (2023). Hysteresis behavior of Auxetic Perforated Steel Plate Shear Walls with elliptical and peanut-shaped cutouts. J. Build. Eng..

[44] Gao Y.T., Chang Y.F., Bai Y., Wang H. (2024). Ultra-wide low-frequency bandgap characteristics of auxeticity-based composite resonator for elastic wave manipulation and machine learning-based inverse structural design. Mater. Today Commun..

[45] Cheng Z.B., Shi Z.F. (2013). Novel composite periodic structures with attenuation zones. Eng. Struct..

[46] Liu Y.F., Huang J.K., Li Y.G., Shi Z.F. (2019). Trees as large-scale natural metamaterials for low-frequency vibration reduction. Constr. Build. Mater..

